# Supplementary material for: Covid-19 vaccine hesitancy and its predictors among diabetic patients on follow-up at public hospitals in Nekemte Town, Western Ethiopia
Source: PLoS One. 2024 Jul 8;19(7):e0305200. doi: 10.1371/journal.pone.0305200 (PMC11230570; doi:10.1371/journal.pone.0305200)
Supplement: Attachment — Submitted filename: My comments.docx [file pone.0305200.s002.docx]

Dear Editor, thank you for allowing me to review this manuscript. This manuscript need major revision. The following are my comments and suggestion.

1. Abstract sections line 23 to 24, what does it mean …………………………by trained nursing working at study institutions (nurses and nursing are different).
2. Abstract section (method subsection)- more information (like the model used, p- value) to analyse should be added
3. Abstract line 31 ~~(Adjusted Odd Ratio [~~AOR~~]~~
4. Abstract line 32: having a great deal of what?
5. Abstract line 33: a fear amount (AOR= 0.046(0.003-0.791)) of awareness on the vaccine preparation ? (not clear for me)
6. Abstract line 34-36: exposure to COVID-19 infection status as strongly agree/agree (AOR = 0.031(0.006-0.171))and neither agree nor disagree (AOR = 36 0.071(0.017-0.295)). How did you specifically see the association between strongly agree/agree and neither agree nor disagree with vaccine hesitancy to the question of ‘’COVID-19 infection status’’? (Likert scale have its own way analysis method).
7. Abstract section-conclusion subsection line 37: The study confirms low rate of COVID-19 vaccine hesitancy……………the term ‘’confirms’’ and ‘’rate’’ should be modified
8. Abstract line 39-42: The relevant agency should focus on efforts to translating these high levels of vaccine acceptance into actual uptake, through targeting identifying determinate factors and vaccine availability for a high-risk diabetes patient. Your result and conclusion contradict each other (low rate of vaccine hesitancy and high level of vaccine acceptance). Grammatically wrong sentences (……..through targeting identifying…………)
9. Most references cited at the end of paragraph throughout the documents. It is recommended to cite after each sentences
10. Introduction line 55 (cite references after ……..COVID-19)
11. Introduction line 58 (cite references after ……SARS-CoV-2 infection)
12. Introduction line 59 (….vaccine hesitancy varying from 76.4% to 3.0%). Better if changed to ….varying from 3.0% to 76.4%
13. Introduction line 67 ( cite references after …….sociodemographic contexts)
14. Introduction line 71- you only cite one reference (8) for two study findings (81.6% in South Africa to 65.2% in Nigeria). How it could be?
15. Again introduction line 71-72 This sentence ‘’In Ethiopia, the hesitancy rate range between 19.1%- 60.3%% were documented in the studies conducted in different part of the country’’ should be cited for each both findings.
16. Introduction line 77-76- repetitions (….among the among high-risk population….)
17. Introduction line 77-80- the last sentences is too long sentence and difficult to catch up the main idea of the sentences. Better if break it down to make mor meaningful and understandable.
18. Line 96-97- what to mean? ‘’All selected diabetic patients (Type I and II) who were attending diabetic clinic of public hospitals in Nekemte Town during the study period’’
19. Exclusion criteria?
20. Line 102-103- why did you use 50% for sample size calculation? You wrote that in introduction section numerous studies reported the hesitancy rate range between 19.1%-60.3%%.
21. Line 105- ‘’A total of 422 patients with known DM participated in the study’’ this sentences should be under result sections (It should be as follows; So, the final sample size was 422).
22. Line 108- from when to when you counted the total number of DM patients on follow-up from the registries?
23. Line 117-119- ‘’ The 118 questionnaire is adapted from different relevant previous studies in the area (2, 4, 10) that adapted 5 119 and modified to suit the current study’’. Should be rewritten
24. Line 124-125 ‘’The questionnaire was administered face to 125 face by trained nursing working in the study facility diabetic clinic or Wards’’. Should be re-written
25. Line 129- add the actual number on which you conducted pre-test
26. Line 143-144- ‘’Variables with a P-values of <0.05 in the bi-variable logistic regression analysis were entered in the multivariable logistic regression analysis to control the possible effect of confounders’’. What is your base to use p-value <0.05 to select your candidate variables in bi-variable logistic regression?
27. What are about multicollinearity and model fitness test?
28. General comments on result sections; Almost you doubled your result section, (you wrote in texts and again you put those variables in the table in almost all of your result sections. If you explain it in text or by paragraph, you are not expected to put those variable in the table form and vice versa.
29. Line 184-185- how did you measure whether the overall health condition of study participants as very poor, poor, average, good and very good?
30. Line 218 to 229- I didn’t like the way the factors were written in all. I strongly recommend the authors to talk and consult the staticians (biostaticians and epidemiologist).
31. Line 230- Table 4: add multivariable logistic regression also on the title
32. In the table: not ‘’income per monthly’’ rather ‘’Monthly income’’
33. Discussion line 270-282- not well discussed.
