# Supplementary material for: Covid-19 vaccine hesitancy and its predictors among diabetic patients on follow-up at public hospitals in Nekemte Town, Western Ethiopia
Source: PLoS One. 2024 Jul 8;19(7):e0305200. doi: 10.1371/journal.pone.0305200 (PMC11230570; doi:10.1371/journal.pone.0305200)
Supplement: Attachment — Submitted filename: Response to reviewers.docx [file pone.0305200.s003.docx]

**Author's response to Reviewers Comments**

PONE-D-23-22981

**Title of Manuscript:** Covid-19 Vaccine Hesitancy and Predictor Factors among Diabetic Patients on Follow-Up at Public Hospitals in Nekemte Town, Western Ethiopia

**Authors:**

1. Aberash Olani Kuta: aberashkuta@gmail.com
2. Nagasa Dida: [nadibefe@yahoo.com](mailto:nadibefe@yahoo.com)

***Generally, in manuscript we have used track change to incorporate or address the reviewers’ comments.***

**A Point by point response**

**Response to ACADEMIC EDITOR**

- The paper has major grammar and language flaws that needs intense revisions. I strongly recommend this paper be edited by subject language experts.
  - **Response from authors:** It is alright. As you indicate there are many errors. Using language expert we tried to correct using track change throughout the manuscript and colored highlight also made.
- Abstract: This section should be concise but informative. Describe aim of the study, and make clearer the sampling strategy, and analysis model and approaches. For results (AOR), limit the decimal points to 2 decimal points and explore the reasons for wider confidence interval.
  - **Response from authors:** Corrected as suggested across the whole document
- Correct the data availability statement. PLOS ONE journal requires authors to supply data used in this paper or clearly indicate the site where the data can be easily accessed. If data are not shared at this time, authors should explain why they are not supplying research data.
  - **Response from authors:** Data set is uploaded as supplementary document
- Rewrite the research ethics statement (the flow of idea is distorted) and describe how the confidentiality of respondents information is maintained.
  - **Response from authors:** reorganized and how confidentiality is maintained also incorporated
- Change the in-text citation format to square brackets (such as: [1])
  - **Response from authors:** Changed
- Introduction: Line # 47-49: How you are sure that existing COVID-19 control measures (including vaccine) had no impact in stopping disease transmission, hospitalization and death? Provide an evidence that supports this. In the first use, describe abbreviations in full terms (e.g., DM, line #57). Try to provide some contextual information about COVID-19 in Ethiopia, including the date it is notified; the burden it had and the different control measures adapted and implemented. Supplement information about the prevailing factors of vaccine hesitancy in Ethiopia. Try to also to review studies that conducted focusing on high-risk patients or population groups.
  - **Response from authors:** Reviewed
- Methods: Describe the contents of the questionnaire, and supply the English version of the tool as supplementary information. How the final regression model fitness was assessed. Have you did multicollinearity test? if so, what is the result?  Why you used P<0.05 in the bivariate regression? Operationalize some terms such as: Overall health.
  - **Response from authors:** Stated as shown using track change
- Results: Correct the interpretations of the findings and make it easily understandable to readers (Line # 219 and afterwards)

**Response to Reviewer #1:**

**RESPONSE TO SPECIFIC COMMENTS:**

1. Abstract sections line 23 to 24, what does it mean …………………………by trained nursing working at study institutions (nurses and nursing are different).

- **Response from the authors:** Sorry, it is to mean trained nurse (nurse who had traing and experience of data collection). Nurse is a person who practice Nursing which is a profession. It has corrected accordingly.

2. Abstract section (method subsection)- more information (like the model used, p- value) to analyse should be added

- **Response from the authors:** Corrected

3. Abstract line 31 (Adjusted Odd Ratio [AOR]
   - **Response from the authors:** Corrected
4. Abstract line 32: having a great deal of what?
   - **Response from the authors:** It was one of a response for a question ‘How much you trust the current COVID19 vaccine development process?’ It means that ‘Having great trust on the vaccines development process’ and now I have made it clear.
5. Abstract line 33: a fear amount (AOR= 0.046(0.003-0.791)) of awareness on the vaccine preparation? (not clear for me)

- **Response from the authors:** It was one of a response for a question ‘How much you trust the current COVID19 vaccine development process?’ I have clarified it now

6. Abstract line 34-36: exposure to COVID-19 infection status as strongly agree/agree (AOR = 0.031(0.006-0.171)) and neither agree nor disagree (AOR = 36 0.071(0.017-0.295)). How did you specifically see the association between strongly agree/agree and neither agree nor disagree with vaccine hesitancy to the question of “COVID-19 infection status” ? (Likert scale have its own way analysis method).
   - **Response from the authors:** Right you are, Likert scale questions have its own analysis approach. In this, it deals with one idea, that is why considered a single explanatory variable. It is about exposure to COVID-19 infection status after having the vaccine. To make it clear for the reader, I have revised it.
7. Abstract section-conclusion subsection line 37: The study confirms low rate of COVID-19 vaccine hesitancy……………the term ‘’confirms’’ and ‘’rate’’ should be modified
   - **Response from the authors:** Modified
8. Abstract line 39-42: The relevant agency should focus on efforts to translating these high levels of vaccine acceptance into actual uptake, through targeting identifying determinate factors and vaccine availability for a high-risk diabetes patient. Your result and conclusion contradict each other (low rate of vaccine hesitancy and high level of vaccine acceptance). Grammatically wrong sentences (……..through targeting identifying…………)
   - **Response from the authors:** low number of patients hesitate vaccine uptake mean that large or high number of patients have willingness to take the vaccine; revision has done.
9. Most references cited at the end of paragraph throughout the documents. It is recommended to cite after each sentences
   - **Response from the authors:** Idea preceding the references was synthesized form that reference. Using same reference for each sentence in the same paragraph might be boring because of redundant use same reference in a single paragraph. That is why we use at the end of the paragraph. Still there are references used in paragraph.
10. Introduction line 55 (cite references after ……..COVID-19)
    - **Response from the authors:** Done
11. Introduction line 58 (cite references after ……SARS-CoV-2 infection)
    - **Response from the authors:** Done
12. Introduction line 59 (….vaccine hesitancy varying from 76.4% to 3.0%). Better if changed to ….varying from 3.0% to 76.4%
    - **Response from the authors:** changed
13. Introduction line 67 (cite references after …….sociodemographic contexts)
    - **Response from the authors:**
14. Introduction line 71- you only cite one reference (8) for two study findings (81.6% in South Africa to 65.2% in Nigeria). How it could be?
    - The study was done by World Health Organization, it addresses many countries that is why findings from the two countries taken from one reference.
15. Introduction line 77-76- repetitions (….among the among high-risk population….)
    - **Response from the authors:** Corrected
16. Introduction line 77-80- the last sentences is too long sentence and difficult to catch up the main idea of the sentences. Better if break it down to make more meaningful and understandable.
    - **Response from the authors:** Done
17. Line 96-97- what to mean? ‘’All selected diabetic patients (Type I and II) who were attending diabetic clinic of public hospitals in Nekemte Town during the study period’’
    - **Response from the authors:** Rephrased, it is to mean the study population.
18. Exclusion criteria?
    - **Response from the authors:** Included
19. Line 102-103- why did you use 50% for sample size calculation? You wrote that in introduction section numerous studies reported the hesitancy rate range between 19.1%-60.3%%.
    - **Response from the authors:** The study population were different, that is why 50% was used.
20. Line 105- ‘’A total of 422 patients with known DM participated in the study’’ this sentences should be under result sections (It should be as follows; So, the final sample size was 422).
    - **Response from the authors:** Corrected as suggested by the reviewer
21. Line 108- from when to when you counted the total number of DM patients on follow-up from the registries?
    - **Response from the authors:** Time frame was not considered. Regardless of time period the patients on the follow up, all those on the registration book was a source population and study population was sampled from it.
22. Line 117-119- ‘’ The 118 questionnaire is adapted from different relevant previous studies in the area (2, 4, 10) that adapted and modified to suit the current study’’. Should be rewritten
    - **Response from the authors:** Re-written**.**
23. Line 124-125 ‘’The questionnaire was administered face to face by trained nursing working in the study facility diabetic clinic or Wards’’. Should be re-written
    - **Response from the authors:** Rewritten
24. Line 129- add the actual number on which you conducted pre-test
    - **Response from the authors:** They were 21 and I have added to the manuscript
25. Line 143-144- ‘’Variables with a P-values of <0.05 in the bi-variable logistic regression analysis were entered in the multivariable logistic regression analysis to control the possible effect of confounders’’. What is your base to use p-value <0.05 to select your candidate variables in bi-variable logistic regression?
    - **Response from the authors:** During the proposal it was planned to use a p-value of 0.25 in order not miss some variable because of the confounding factors. But, during the analysis practically we found all the variable with p-value of 0.05, that is why 0.05 was stated.
26. What are about multicollinearity and model fitness test?
    - **Response from the authors:** To check presence of multicollinearity among the independent variables Variance Inflation Factors with a cutoff point 10 and Hosmer Lemeshow goodness of fit test was used and we found that its p-value was greater than 0.05. If mandatory, we can include in the analysis section.
27. General comments on result sections; Almost you doubled your result section, (you wrote in texts and again you put those variables in the table in almost all of your result sections. If you explain it in text or by paragraph, you are not expected to put those variable in the table form and vice versa.
    - **Response from the authors:** Revised
28. Line 184-185- how did you measure whether the overall health condition of study participants as very poor, poor, average, good and very good?
    - **Response from the authors:** Here standard tool was not used, it just personal feeling and reflection of the patients.
29. Line 218 to 229- I didn’t like the way the factors were written in all. I strongly recommend the authors to talk and consult the staticians (biostaticians and epidemiologist).
    - **Response from the authors:** ???
30. Line 230- Table 4: add multivariable logistic regression also on the title
    - **Response from the authors:** Done
31. In the table: not ‘’income per monthly’’ rather ‘’Monthly income’’
    - **Response from the authors:** Corrected
32. Discussion line 270-282- not well discussed.
    - **Response from the authors:** Re-discussed

**Reviewer #2: Tittle:**
• Change the title to "COVID-19 Vaccine Hesitancy and its Predictors among Diabetic Patients on Follow-Up at Public Hospitals in Nekemte Town, Western Ethiopia."

- **Response from the authors:** Changed

Abstract
• Line 25, “Statically” to “statistical”.

- **Response from the authors:** Corrected

Introduction
• The gaps weren't stated well, especially in relation to patients with diabetes, even though the introduction was written well. Three key considerations need to be made in this instance;
♣ Principles and practices
♣ Gaps, which discuss what was and wasn't known
♣ Filling the gaps

- **Response from the authors:** Revised

Methods and materials
• Line 96-97, “All selected diabetic patients (Type I and II) who were attending diabetic clinic of public hospitals in Nekemte Town during the study period”. What this statement indicates? Who are your study populations?

- **Response from the authors:** The study population were those patients selected by systematic random sampling. I have re-written

• Why you have considered P=50% for sample size calculation? Justification?

- **Response from the authors:** During that study period**, t**here was no similar study on similar population, that is why 50% was used.

• How you have calculated K (8) value for both hospital and how it could be? Clarify the proportional allocation of the sample to each study hospitals.

- **Response from the authors:** To get ‘k’, all DM patients on follow at the health facility were added together and divided for the sample. The proportion was also done based on number of DM patients on follow-up at each health facility.

• Line 118, rephrase ”The questionnaire is adapted from different relevant previous studies in the area (2, 4, 10) that adapted and modified to suit the current study”

- **Response from the authors:** Rephrased

• Line 120 “Afan Oromo (local language)” to “Afan Oromo” omitting local language

- **Response from the authors:** Rephrased

• Have you actually verified the questionnaire's validity? What was your proof, if any?

- **Response from the authors:** Corrected as suggested by the reviewer

• Be consistent while using the terms like predictors, factors and determinant.

- **Response from the authors:** Corrected

**Result**

• The phrase "In this study" appears multiple times in lines 161,169, 172 and etc. in the manuscript. Put it in a different way.

- **Response from the authors:** Corrected

• Line 164 “>=50” to “>50” and also in table 1

- **Response from the authors:** Even though all categories of a given variable is not described in the description section, the one described and not have to present in the table. That is why it exist both in the narrative part and table as well. If I understood and responded negatively, let you elaborate more so that I will explain more.

• Line 174 Clinical Characteristics study participants to “Study participants' clinical characteristics”

- **Response from the authors:** Corrected as suggested by the reviewer

• Clearly define any terms. As stated in Table 2, the DM diseased year, DM type, and overall health

- **Response from the authors:** done

• Line 202, less the one-third? Means?

- **Response from the authors:** The frequency is incorporated for a clarity

• In table 3, you have stated that as” Tested positive for COVID-19 - --17(4.0%) and Family died of COVID-19---17(4.0%).What this indicate??

- **Response from the authors:** It is simply to presents information about the participants' experiences with COVID-19 exposure and testing, and COVID-19 related death in the family.

• Table 3 reported that 235 individuals (55.7%) had received the COVID-19 vaccine; however, the overall rate of vaccine hesitancy was 15.2%. This suggests that 84.8% of people do not have vaccine hesitancy. Conversely, what can we say about those 29.1% in this regard?

- **Response from the authors:** Those patients who have already took the vaccine and those are ready to take but haven’t took yet considered as they have no vaccine hesitancy. Moreover, from those who haven’t received yet and has no willingness to take the vaccine were counted under vaccine hesitancy. Hence, the 29.1% are those have plan and waiting to take the vaccine.

• Line 220, “The study participants who have vaccine awareness had 0.029 less odd to hesitate (AOR= 0.029(0.001222 0.857)”. Rephrase it and how you have described the odd ratio was not clear. Please apply this concern to all.

- **Response from the authors:** Rephrased

**Discussion**
• As per your objectives, the discussion section ought to center around the relevant findings that emerged from the result section. Modifications are necessary to the way it has been discussed.

- **Response from the authors:** Modification has made revised

**Conclusion**
• I felt that your findings and recommendation in this section were in conflict with one another. Your recommendation should therefore be based on your relevant findings and should be appropriate.

- **Response from the authors: Corrected**

**Generally:**
• The manuscript needs to be re-narrated in clear, concise English, with consistency and coherence in the use of terminology

- **Response from the authors:** Revision has mad across the whole document
